# Numerical Stability of Partitioned Approach in Fluid-Structure Interaction for a Deformable Thin-Walled Vessel

**DOI:** 10.1155/2013/638519

**Published:** 2013-10-08

**Authors:** Kelvin K. L. Wong, Pongpat Thavornpattanapong, Sherman C. P. Cheung, Jiyuan Tu

**Affiliations:** School of Aerospace, Mechanical & Manufacturing Engineering, RMIT University, P.O. Box 71, Bundoora, VIC 3083, Australia

## Abstract

Added-mass instability is known to be an important issue in the partitioned approach for fluid-structure interaction (FSI) solvers. Despite the implementation of the implicit approach, convergence of solution can be difficult to achieve. Relaxation may be applied to improve this implicitness of the partitioned algorithm, but this commonly leads to a significant increase in computational time. This is because the critical relaxation factor that allows stability of the coupling tends to be impractically small. In this study, a mathematical analysis for optimizing numerical performance based on different time integration schemes that pertain to both the fluid and solid accelerations is presented. The aim is to determine the most efficient configuration for the FSI architecture. Both theoretical and numerical results suggest that the choice of time integration schemes has a significant influence on the stability of FSI coupling. This concludes that, in addition to material and its geometric properties, the choice of time integration schemes is important in determining the stability of the numerical computation. A proper selection of the associated parameters can improve performance considerably by influencing the condition of coupling stability.

## 1. Introduction

Fluid-structure interaction (FSI) is used widely in biomechanical computer simulations. The modelling of pulsatile blood flow in elastic vessels requires a framework that can handle the blood-vessel interaction, and the implementation of FSI can solve the time dependent biofluid flow through its elastic structure. Useful information such as the severity of vessel damage by abnormal flow, degree of plaque growth or risk of its rupture in diseased arteries, and the aggravation of atherosclerosis can be generated for medical evaluation. In general, FSI is an architecture processing the interaction of a solid structure with a dynamic fluid that can be implemented by the monolithic and partitioned approaches. A configured FSI solver can use the former approach to solve a system of governing equations for the fluid and solid domains [1]. Although FSI gives strong coupling between the two domains, the limitations are as follows:

- demanding computational power for solving a large system of equations;
- need for further development of preconditioning;
- lack of specialized capabilities that pertain to “legacy software” such as ABAQUS and ANSYS.

The partitioned approach is attractive due to its advantage of having software modularity that allows selection of an appropriate solver among the well-established solvers for each of the domains. Nevertheless, its efficiency is inferior to its monolithic counterpart due to the existence of an “added-mass instability,” which commonly occurs in problems involving large deformation and light weight structure. This causes divergence and failure before the final solution can be achieved. In order to handle this issue, small values for coupling relaxation factors of interface loads must be used in order to maintain stability. However, that will lead to a significant increase in computational time. As such, much work has been performed to search for techniques that can deal with this instability. For example, adaptive relaxation techniques that are based on using information of earlier iterations for approximating an appropriate relaxation value and providing stability have been employed to increase the speed of calculation [2]. It is well understood that FSI solution experiences this numerical instability when the following conditions [3] are observed:

- stability of FSI solution tends to be critically severe when density ratio of fluid to solid is excessively high;
- increase in fluid viscosity leads to a decrease in stability of the FSI solver, and a corresponding increase in structural stiffness improves this stability;
- temporal discretisation schemes used for FSI calculation can influence the instability condition;
- decrease in time step size used for FSI calculation can give an earlier occurrence of its instability.

The observed behaviors of instability can be explained mathematically when conditions for stability of both explicit and implicit FSI solution of a flexible cylindrical vessel are demonstrated [4]. However, the impact of time integration schemes of the fluid and solid accelerations that is used in the FSI calculation is still not fully understood and therefore justifies the need for a thorough investigation in this paper. The aim of this work is to analyze performance of FSI using a partitioned approach based on different time integration schemes that pertain to the structural mechanics. We conduct the analysis on a simplified problem of pressure wave propagation along a flexible cylindrical vessel. Influence of the parameters relating to the FSI performance can be summarized as follows:

- time integration schemes for solid and fluid accelerations;
- time step size;
- ratio of fluid to solid densities.

We explore the influence of time integration schemes on a partitioned approach for fluid-structure interaction problems by organising our work in a logical manner. In Section 2, we present a mathematical description of a simplified pressure wave propagation along a flexible cylindrical vessel, fundamental FSI conditions, and the discretization schemes used for deriving the results.  Section 3 provides a mathematical analysis of the stability of implicit FSI based on the different time integration schemes that are discussed. In Section 4, numerical validations and results of the same problem (that are used in Section 3) are demonstrated in order to confirm the validity of our theoretical proofs. Finally, Section 5 summarizes the influence of the time integration schemes and the associated parameters on the stability of the FSI.

## 2. Mathematical Background of Fluid-Structure Interaction

### 2.1. Governing Equations

**Figure 1:**
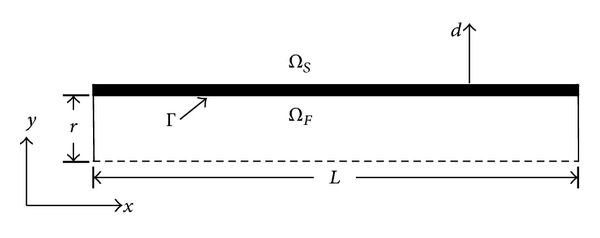
Schematic representation of the computational domain for a flexible vessel transporting fluid.

 A simplified flexible cylindrical tube of radius *r*, length *L*, thickness *h*_*s*_, density *ρ*_*s*_, Young's modulus *E*, and Poisson's coefficient *ν* is chosen for our mathematical analysis. It allows our mathematical and numerical examination to be performed and provides sufficient information for conducting a realistic simulation. We define a fluid domain *Ω*_*F*_ and a structural domain *Ω*_*S*_ that interact at the common boundary Γ. Deformation of cylindrical tube is allowed only in the radial and longitudinal directions. Inlet and outlet of the fluid domain are subjected to pressure boundary conditions. The schematic representation of our computational domain for a fluid through the tube is shown in Figure 1. Note that the dashed lines represent the axis of symmetry of the tube.

#### 2.1.1. Solid Governing Equation

We refer to the governing equation of deformation for a flexible cylindrical tube in [4] as follows:

(1) $$\begin{array}{c} \rho_{s}h_{s}\frac{\partial^{2}d_{\Gamma }}{\partial t^{2}}+a_{0}d_{\Gamma }-b\frac{\partial^{2}d_{\Gamma }}{\partial x^{2}}=P_{\Gamma }, \end{array}$$

where *a*_0_ = *Eh*_*s*_/*r* ^2^(1 − *ν* ^2^), *b* = *K*_*T*_ *Bh*_*s*_. Note that *K*_*T*_ denotes the Timoshenko shear correction factor, *P*_Γ_ is structural load on the interface Γ due to the external forcing term from the fluid, *d*_Γ_ is displacement at the interface, *x* is position in space, and *t* is position in time.

#### 2.1.2. Fluid Governing Equations

Considering a general variable property per unit mass that is denoted as *ϕ*, the generic form of fluid governing equations in an Arbitrary Lagrangian-Eulerian (ALE) frame of reference is stated as

(2) $$\begin{array}{c} \underset{\text{Temporal}\;\text{term}}{\underset{︸}{\frac{\partial \rho \phi }{\partial t}}}+\underset{\text{Convection}\;\text{term}}{\underset{︸}{\nabla \cdot \left(\rho \left[v-v_{c}\right]\phi \right)}}-\underset{\text{Diffusion}\;\text{term}}{\underset{︸}{\nabla \cdot \left(\rho \Upsilon_{\phi }\nabla \phi \right)}}=\underset{\text{Source}\;\text{term}}{\underset{︸}{S_{\phi }\left(\phi \right)}}. \end{array}$$

In the finite volume method, it is required that the governing equation is satisfied over the control volume **V**_*P*_ around a point *P*. Therefore, it can be rewritten in the integral form as

(3) ∫tt+Δt{∂∂t∫VPρϕ dVP︸Temporal  term+∫VP∇·ρ([v−vc]ϕ)dVP︸Convection  term−∫VP∇·(ρΥϕ∇ϕ)dVP︸Diffusion  term}dt=∫tt+Δt{∫VPSϕ(ϕ)dVP︸Source  term}dt.

#### 2.1.3. The Effective FSI Governing Equation

In order to analyze stability of FSI calculation, an effective FSI governing equation can be expressed as

(4) $$\begin{array}{c} \rho_{s}h_{s}\frac{\partial^{2}d_{\Gamma }}{\partial t^{2}}+a_{0}d_{\Gamma }-b\frac{\partial^{2}d_{\Gamma }}{\partial x^{2}}=P_{\Gamma }. \end{array}$$

The structural load that is influenced by the fluid load *P*_ext,Γ_ is given by

(5) $$\begin{array}{c} P_{\Gamma }=P_{\text{ext},\Gamma }-\rho_{f}M_{a}\frac{\partial^{2}d_{\Gamma }}{\partial t^{2}}, \end{array}$$

where *M*_*a*_ denotes the added-mass operator matrix.

By coupling the two domains, we can achieve the interaction of the fluid and structure based on

(6) $$\begin{array}{c} \rho_{s}h_{s}\frac{\partial^{2}d_{\Gamma }}{\partial t^{2}}+\rho_{f}M_{a}\frac{\partial^{2}d_{\Gamma }}{\partial t^{2}}+a_{0}d_{\Gamma }-b\frac{\partial^{2}d_{\Gamma }}{\partial x^{2}}=P_{\text{ext},\Gamma }. \end{array}$$

Since we are interested in studying the added-mass instability where the mass term dominates the stiffness term, some nonlinearity is neglected. This results in

(7) $$\begin{array}{c} \rho_{s}h_{s}\frac{\partial^{2}d_{\Gamma }^{s}}{\partial t^{2}}+\rho_{f}M_{a}\frac{\partial^{2}d_{\Gamma }^{f}}{\partial t^{2}}+a_{0}d_{\Gamma }^{s}=P_{\text{ext},\Gamma }. \end{array}$$

### 2.2. Fundamental FSI Coupling Conditions

The partitioned approach can enable the physical integration of the fluid and solid domains that is demanded by the simulation of flow through an elastic vessel. The fluid-structure interface is enforced by iteration between the structural and fluid physics modules until convergence is reached. The arbitrary mesh motion at discrete time steps can be computed by the timeintegration of the set of partial-differential equations in Section 2.1 for both the solid and fluid structures that govern the mesh motion.

The conditions required when solving FSI pertain to the kinematic and dynamic nature as suggested by numerous works (see [5] and reference therein). The kinematic condition ensures the compatibility of displacement across FSI interface and can be written as

(8) $$\begin{array}{c} d_{\Gamma }^{f}=d_{\Gamma }^{s}, \end{array}$$

where *d* is displacement, *d*_Γ_^*s*^ and *d*_Γ_^*f*^ are the displacements of the solid and fluid interface, respectively.

Assuming that if no-slip condition is used on the fluid side of the FSI interface, this condition leads to a relationship between fluid velocity **v**_Γ_ and rate of change of displacement, which can be written as

(9) $$\begin{array}{c} \hat{n}\cdot u_{\Gamma }=\hat{n}\cdot {\dot{d}}_{\Gamma }^{s}. \end{array}$$

The dynamic condition ensures the compatibility of traction across FSI interface and gives rise to

(10) $$\begin{array}{c} \hat{n}\cdot \tau_{\Gamma }^{s}=\hat{n}\cdot \tau_{\Gamma }^{f}, \end{array}$$

where *τ*_Γ_ represents stress on the interface.

These two conditions are normally utilized in FSI codes that adopt the partitioned approach. By implementation of the kinematic condition, fluid nodes on the FSI interface are updated according to their corresponding solid nodes. By doing the same for the dynamic condition, the equilibrium of stress on FSI interface is ensured and the fluid pressure is integrated into a fluid force, which is used in applying to the solid nodes along the interface.

### 2.3. Time Integration Schemes

#### 2.3.1. Discretization of Structural Acceleration

The nonlinear version of the generalized-*α* time integration scheme that was introduced by [6] can be used for discretization of solid governing equation [7] and is given by

(11) $$\begin{array}{c} {\dot{d}}^{n+1}={\dot{d}}^{n}+\left[\left(1-\delta \right){\ddot{d}}^{n}+\delta {\ddot{d}}^{n+1}\right]\Delta t,\\ d^{n+1}=d^{n}+{\dot{d}}^{n}\Delta t+\left[\left(\frac{1}{2}-\alpha \right){\ddot{d}}^{n}+\alpha {\ddot{d}}^{n+1}\right]\Delta t^{2}, \end{array}$$

where Δ*t* denotes the discrete time step interval.

After some mathematical manipulations, (11) can be recasted into the following equations as:

(12) $$\begin{array}{c} {\dot{d}}^{n+1}={\dot{d}}^{n}+\Delta t\left(1-\delta \right){\ddot{d}}^{n}+\Delta t\delta {\ddot{d}}^{n+1},\\ {\ddot{d}}^{n+1}=\frac{1}{\alpha \Delta t^{2}}\left(d^{n+1}+d^{n}\right)-\frac{1}{\alpha \Delta t}{\dot{d}}^{n}-\left(\frac{1}{2\alpha }-1\right){\ddot{d}}^{n}. \end{array}$$

The value of structural displacement, velocity, and acceleration is interpolated between time level as

(13) $$\begin{array}{c} {\ddot{d}}^{n+1,\alpha }=\left(1-\alpha_{m}\right){\ddot{d}}^{n+1}+\alpha_{m}{\ddot{d}}^{n},\\ {\dot{d}}^{n+1,\alpha }=\left(1-\alpha_{f}\right){\dot{d}}^{n+1}+\alpha_{f}{\dot{d}}^{n},\\ d^{n+1,\alpha }=\left(1-\alpha_{f}\right)d^{n+1}+\alpha_{f}d^{n}. \end{array}$$

In order to maintain unconditional stability and second order accuracy of time integration, the following criteria must be satisfied [6]:

(14) $$\begin{array}{c} \delta \geq \frac{1}{2},\;\;\alpha \geq \frac{1}{2}\delta ,\;\;\delta =\frac{1}{2}-\alpha_{m}+\alpha_{f},\\ \alpha_{m}\leq \alpha_{f}\leq \frac{1}{2}. \end{array}$$

For numerical damping, [8] suggests that these parameters can be written in terms of amplitude decay factor *γ* as

(15) $$\begin{array}{c} \alpha =\frac{1}{4}{\left(1+\gamma \right)}^{2},\;\;\delta =\frac{1}{2}+\gamma ,\\ \alpha_{f}=0,\;\;\alpha_{m}=-\gamma . \end{array}$$

When minimum numerical damping is applied, the values of *α*, *δ*, *α*_*m*_, and *α*_*f*_ are set to be 1/4, 1/2, 0, and 0, respectively. These values represent time integration that has zero numerical damping, which can be achieved by setting *γ* = 0. The equations for ${\ddot{d}}^{n+1}$ and ${\dot{d}}^{n+1}$ can be stated as

(16) $$\begin{array}{c} {\dot{d}}^{n+1}={\dot{d}}^{n}+\frac{1}{2}\Delta t{\ddot{d}}^{n}+\frac{1}{2}\Delta t{\ddot{d}}^{n+1},\\ {\ddot{d}}^{n+1}=\frac{4}{\Delta t^{2}}\left[d^{n+1}+d^{n}-\Delta t{\dot{d}}^{n}-\frac{\Delta t^{2}}{4}{\ddot{d}}^{n}\right]. \end{array}$$

After mathematical manipulation, the structural acceleration can be written in terms of deformation at different time levels as

(17) d¨n+1,α≈1Δt2(4dn+1−16dn+32dn−1−20dn−2) −12Δtd˙n−2−d¨n−2.

This time integration scheme cannot be put in terms of displacement only, and it has the fully recursive characteristics, which means that the calculation of time step *n* + 1 utilizes information of all previous time steps down to the intial step.

When maximum numerical damping is applied, the values of *α*, *δ*, *α*_*m*_, and *α*_*f*_ are set to be 1, 3/2, −1, and 0, respectively. This can be achieved alternatively by setting *γ* = 1. Therefore, equations for ${\ddot{d}}^{n+1}$ and ${\dot{d}}^{n+1}$ can be written as

(18) $$\begin{array}{c} {\dot{d}}^{n+1}={\dot{d}}^{n}+\frac{1}{2}\Delta t{\ddot{d}}^{n}+\frac{3}{2}\Delta t{\ddot{d}}^{n+1},\\ {\ddot{d}}^{n+1}=\frac{1}{\Delta t^{2}}\left[d^{n+1}+d^{n}-\Delta t{\dot{d}}^{n}-\frac{\Delta t^{2}}{2}{\ddot{d}}^{n}\right]. \end{array}$$

Subsequently, this leads to

(19) $$\begin{array}{c} {\ddot{d}}^{n+1,\alpha }=\frac{1}{\Delta t^{2}}\left(2d^{n+1}-5d^{n}+4d^{n-1}-d^{n-2}\right). \end{array}$$

#### 2.3.2. Discretization of Fluid Acceleration

 Two backward Euler schemes are used for the discretization of fluid acceleration. The first order backward Euler scheme can be written as

(20) $$\begin{array}{c} \frac{u^{n+1}-u^{n}}{\Delta t}={\dot{u}}^{n+1}, \end{array}$$

while the second order backward Euler scheme is

(21) $$\begin{array}{c} \frac{u^{n+1}-u^{n}}{\Delta t}=\frac{1}{3}\frac{u^{n}-u^{n-1}}{\Delta t}+\frac{2}{3}{\dot{u}}^{n+1}. \end{array}$$

In this paper, only the zeroth order structural predictor is used in order to estimate the fluid deformation corresponding to structural displacement of previous time step *d* ^*n*^. This structural predictor can be written as

(22) $$\begin{array}{c} d_{\Gamma ,P}^{n+1}=d_{\Gamma }^{n}. \end{array}$$

## 3. Mathematical Analysis

A stability analysis of FSI calculation based on implicit coupling is presented. By adopting this implicit coupling, several iterations are needed for each time step, and relaxation is applied to maintain the stability of calculation.

### 3.1. Numerical Procedure of Implicit Coupling

An interface code processes the data transfer between the fluid and solid domains and mesh association across their respective processors. The following steps are used for achieving implicit coupling. Initial guess *d*_0_^*n*+1^ is given and coupling iteration *m* = {0,1, 2,3,…}.

(1) Estimate interface deformation according to previous time or coupling step by using (22), and update the interior fluid mesh.
(2) Execute fluid solver, *F*(*d*), such that
  (23) $$\begin{array}{c} {\tilde{P}}_{m+1}^{n+1}=F\left({\tilde{d}}_{m+1}^{n+1}\right). \end{array}$$
(3) Execute solid solver, *S*(*P*), such that
  (24) $$\begin{array}{c} {\tilde{d}}_{m+1}^{n+1}=S\left({\tilde{P}}_{m+1}^{n+1}\right). \end{array}$$
(4) Apply relaxation such that
  (25) $$\begin{array}{c} d_{m+1}^{n+1}=\left(1-\omega \right){\tilde{d}}_{m+1}^{n+1}+\omega d_{m}^{n+1}. \end{array}$$
  Note that the critical relaxation factor is denoted by *ω*.
(5) Check convergence. The solution is converged if the following conditions are applied:
  (26) $$\begin{array}{c} r^{n+1,s}<\varepsilon_{0},\;r^{n+1,s}=d_{m+1}^{n+1}-d_{m}^{n+1},\\ r^{n+1,f}<\varepsilon_{0},\;r^{n+1,f}=P_{m+1}^{n+1}-P_{m}^{n+1}. \end{array}$$

### 3.2. Stability Condition When Minimum Numerical Damping Is Applied

In this section, we discuss the analysis of explicit FSI coupling when minimum numerical damping is implemented. The discretized forms of the structural acceleration and fluid acceleration are given by

(27) d¨Γn+1,s≈1Δt2(4dΓn+1−16dΓn+32dΓn−1−20dΓn−2) −12Δtd˙Γn−2−d¨Γn−2,d¨Γn+1,f=1Δt2(d~Γn+1−2dΓn+dΓn−1).

Contrary to explicit coupling, the latest information used for calculation of FSI interface acceleration of fluid domain comes from the same time level as the one that is used for the structural acceleration. This is due to the application of relaxation factor. Then, discretization of (7) is achieved by substituting (27) to give

(28) ρshsΔt2(4d~Γn+1−16dΓn+32dΓn−1−20dΓn−2−12Δt3d˙Γn−2−1Δt2d¨Γn−2)  +ρfMaΔt2(d~Γn+1−2dΓn+dΓn−1)  +a0dΓn+1=Pext,Γn+1.

For simplification, the analysis can be done by considering only one eigenvector, **v**_*i*_, of solution on the interface. This is because the added-mass operator *M*_*A*_ is a real positive matrix. Therefore, *d*_Γ_ = Σ*d*_*i*_ **v**_*i*_. Notice that the added-mass operator *M*_*A*_ can be represented by the *i*th eigenvalues of *M*_*A*_, *μ*_*i*_. This results in

(29) ρshsΔt2(4d~in+1−16din+32din−1−20din−2−12Δt3d˙in−2−1Δt2d¨in−2)⋯  +ρfμiΔt2(d~in+1−2din+din−1)  +a0din+1=Δt2ρshsPext,in+1.

By substituting (25) into (29), we obtain

(30) ρshsΔt2(4din+1ω−16din+32din−1−20din−2−12Δt3d˙in−2−1Δt2d¨in−2)+a0dm+1,in+1ω⋯ =(1−ωω)(2ρshsΔt2+a0)dm,in+1−ρfμiΔt2(dm,in+1−2din+din−1)+Δt2ρshsPext,in+1.

By means of Von Neumann stability analysis [9],

(31) 1ω(4ρshsΔt2+a0)dm+1,in+1=[(1−ωω)(4ρshsΔt2+a0)−ρfμiΔt2]dm,in+1⋯ +g(din,din−1,din−2,d˙in−2,d¨in−2,Pext,in+1),dm+1,in+1dm,in+1≈[((1−ω)/ω)(4ρshs/Δt2+a0)−ρfμi/Δt2](1/ω)(4ρshs/Δt2+a0)=1−ω(4ρshs+a0Δt2+ρfμi)4ρshs+a0Δt2.︸Growth factor, G

The absolute value of the growth factor has to be less than unity for the solution to be stable. That is,

(32) $$\begin{array}{c} \left|G\right|=\left|1-\frac{\omega \left(4\rho_{s}h_{s}+a_{0}\Delta t^{2}+\rho_{f}\mu_{\max}\right)}{4\rho_{s}h_{s}+a_{0}\Delta t^{2}}\right|<1, \end{array}$$

(33) $$\begin{array}{c} 0<\omega <\frac{8\rho_{s}h_{s}+2a_{0}\Delta t^{2}}{4\rho_{s}h_{s}+a_{0}\Delta t^{2}+\rho_{f}\mu_{\max}}. \end{array}$$

From (33), we see that not only does the material and geometrical properties (such as fluid and solid densities, Young's modulus of the structure, and the maximum eigenvalue of added mass matrix) influence the allowable relaxation factor, but they also affect the time step size of the calculation. It can be further deduced that if the time step size is significantly small and approaches zero, (33) becomes

(34) $$\begin{array}{c} 0<\omega <\frac{8}{4+\rho_{f}\mu_{\max}/\rho_{s}h_{s}}. \end{array}$$

This means that if the time step size is sufficiently small, the Young modulus is no longer a factor that determines the criteria for stability of an implicit algorithm. Therefore, the critical value of relaxation factor converges and does not vary with a further decrease in time step size. Moreover, it can also be concluded that if *ρ*_*f*_ *μ*_max⁡_ = *ρ*_*s*_ *h*_*s*_, the relaxation factor has to be strictly less than 8/5 to allow convergence of solution.

#### 3.2.1. Stability Condition When Maximum Numerical Damping Is Applied

The discretized forms of structural acceleration and fluid acceleration are

(35) $$\begin{array}{c} {\ddot{d}}_{\Gamma }^{n+1,s}=\frac{1}{\Delta t^{2}}\left(2{\tilde{d}}_{\Gamma }^{n+1}-5d_{\Gamma }^{n}+4d_{\Gamma }^{n-1}-d_{\Gamma }^{n-2}\right),\\ {\ddot{d}}_{\Gamma }^{n+1,f}=\frac{1}{\Delta t^{2}}\left({\tilde{d}}_{\Gamma }^{n+1}-2d_{\Gamma }^{n}+d_{\Gamma }^{n-1}\right). \end{array}$$

Unlike explicit coupling, the latest information used for calculation of FSI interface acceleration of fluid domain comes from the same time level as that used for structural acceleration. This is due to the application of the relaxation factor. The discretized form of (7) is achieved by substituting (35) and (45) to give

(36) ρshsΔt2(2d~Γn+1−5dΓn+4dΓn−1−dΓn−2)  +ρfMaΔt2(d~Γn+1−2dΓn+dΓn−1)+a0dΓn+1 =Pext,Γn+1,

(37) ρshsΔt2(2d~in+1−5din+4din−1−din−2)  +ρfμiΔt2(d~in+1−2din+din−1)+a0din+1 =Δt2ρshsPext,in+1.

By substituting (25), (37) can be written as

(38) ρshsΔt2(2dm+1,in+1ω−5din+4din−1−din−2)+a0dm+1,in+1ω =(1−ωω)(2ρshsΔt2+a0)dm,in+1  −ρfμiΔt2(dm,in+1−2din+din−1)+Δt2ρshsPext,in+1,1ω(2ρshsΔt2+a0)dm+1,in+1 =[(1−ωω)(2ρshsΔt2+a0)−ρfμiΔt2]dm,in+1  +g(din,din−1,din−2,Pext,in+1).

By means of Von Neumann stability analysis,

(39) dm+1,in+1dm,in+1≈[((1−ω)/ω)(2ρshs/Δt2+a0)−ρfμi/Δt2](1/ω)(2ρshs/Δt2+a0)=1−ω(2ρshs+a0Δt2+ρfμi)2ρshs+a0Δt2︸Growth factor, G.

Absolute value of the growth factor *G* has to be less than unity if the solution is to be stable. That is,

(40) $$\begin{array}{c} \left|G\right|=\left|1-\frac{\omega \left(2\rho_{s}h_{s}+a_{0}\Delta t^{2}+\rho_{f}\mu_{\max}\right)}{2\rho_{s}h_{s}+a_{0}\Delta t^{2}}\right|<1, \end{array}$$

(41) $$\begin{array}{c} 0<\omega <\frac{4\rho_{s}h_{s}+2a_{0}\Delta t^{2}}{2\rho_{s}h_{s}+a_{0}\Delta t^{2}+\rho_{f}\mu_{\max}}. \end{array}$$

From (41), the material and geometrical properties such as the densities of both the fluid and solid, Young's modulus, and eigenvalue of added-mass matrix have influence on both the allowable relaxation factor and also the time step size of the calculation. This is similar to the case when the minimum numerical damping is applied. As the time step size approaches zero, (41) becomes

(42) $$\begin{array}{c} 0<\omega <\frac{4}{2+\rho_{f}\mu_{\max}/\rho_{s}h_{s}}. \end{array}$$

Here, it is proven that if time step size is very small, the Young modulus is no longer a factor determining the criteria for stability of implicit algorithm. This also means that the critical value of relaxation factor converges and does not vary with a further decrease in time step size. Moreover, it can also be concluded that if *ρ*_*f*_ *μ*_max⁡_ = *ρ*_*s*_ *h*_*s*_, the relaxation factor has to be strictly less than 4/3 to allow convergence of solution.

## 4. Numerical Results

### 4.1. Validation of Computational Model

 Pressure pulse velocity has been widely used as an indicator of blood vessel health. In the past, only physical experiment and analytical solutions were available for obtaining the pulse velocity. Although these analytical solutions can only be used for simple geometries of blood vessel, they can serve as solid verification for numerical simulation, which are becoming popular nowadays. Therefore, a simulation of fluid-structure interaction for calculating pressure pulse velocity in a compliant vessel is conducted to verify our proposed technique in order to compare with analytical solutions such as the Moens-Korteweg equation. The results presented in this section are obtained by using the same method as that used in the next section where numerical results are presented to confirm our theoretical results.

The geometrical model of a three-dimensional tube is shown in Figure 1. The cylindrical domain has a radius of *R*_*s*_ = 0.0005 m and a total length of *L* = 0.06 m. The working fluid is modelled as incompressible fluid with fluid viscosity and density of *μ* = 0.01 Pa·s and *ρ*_*f*_ = 1,000 kg·m^3^, respectively. On the other hand, the compliant vessel is modelled as isotropic material with Poisson's ratio of *ν* = 0.3 and density of *ρ*_*s*_ = 1,000 kg·m^3^, respectively. Young's modulus of the solid structure is varied between *E* = 50 and 200 kPa.

**Figure 2:**
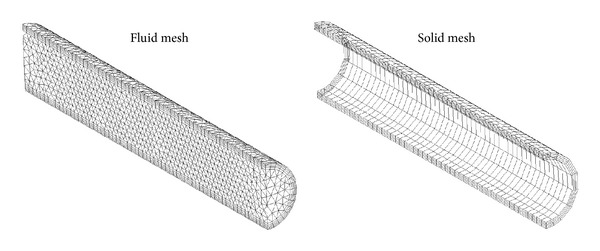
Computational meshes of the fluid and solid domains for a flexible vessel.

Figure 2 shows the computational mesh of both solid and fluid domains that consist of 1,800 and 24,674 elements, respectively. In the fluid mesh, the laminar boundary layer has 5 layers of thin hexagonal elements, while 4 layers are used for the thickness of solid domain. The mesh in the fluid domain is Geometric Conservation Law (GCL) compliant and the architecture generates arbitrary mesh motion at discrete time steps. Now, our main aim is to validate the theoretical results.

Then, analytical sets of equations developed for pressure wave velocity are discussed next. The Moens-Korteweg equation [10, 11] is

(43) $$\begin{array}{c} c=\sqrt{\frac{Eh_{s}}{2R_{s}\rho_{s}}}, \end{array}$$

where *c* represents the pressure wave velocity.

The Wylie and Streeter equation [12] is

(44) $$\begin{array}{c} c=\varphi c_{f}^{*}, \end{array}$$

where *φ* is the stress factor and *c*_f_* is the corrected pressure wave velocity as given by

(45) φ=1−ν2[1−EK(hs2r)(1−KρfEρs)]−1,cf∗=Eρf((2Rs+2hs)2hs(2r+hs)−2(1−ν))−1.

**Figure 3:**
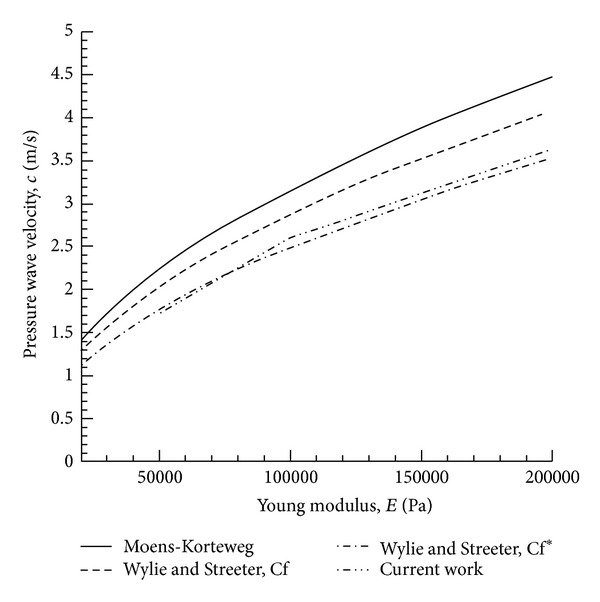
Relationship between pressure wave velocity and Young modulus of a flexible vessel.

Note that *K* is the bulk modulus of elasticity of the tube walls. These two equations will be utilised for the validation of numerical method used in this work. The relationship between pressure wave velocity and Young's modulus of flexible vessel illustrates adequate accuracy of the method (Figure 3).

**Figure 4:**
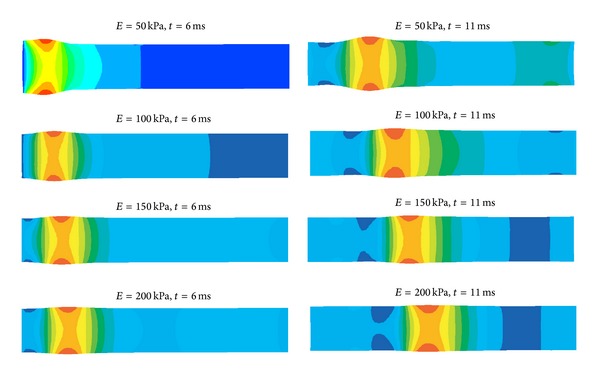
Pressure wave propagation along a flexible cylindrical vessel at various Young moduli and time levels.

The pressure pulse velocity is calculated by measuring the location of maximum deformation at two different times based on the sixth and eleventh millisecond. Distance between the two locations is divided by a time difference of five milliseconds in order to obtain the velocity of the pressure pulse. This location is used for the measurement as it represents the centre of the pressure wave.  Figure 4 shows the contour plot of pressure wave propagation along the flexible cylindrical vessel at different Young's moduli and simulation time levels.

Next, we present a set of numerical results with different structural time integration scheme by varying the amplitude decay factor *γ*. These results can be used to confirm our theoretical experiments. By our default configuration, which is used to as a reference to compare with other cases, we set vessel radius *R*_*s*_ = 0.005 m, vessel length *L* = 0.06 m, vessel thickness *h*_*s*_ = 0.001 m, solid density *ρ*_*s*_ = 30,000 kg·m^3^, Young's modulus *E* = 750,000 Pa, Poisson's ratio *ν* = 0.5, fluid density *ρ*_*f*_ = 1,000 kg·m^3^, and fluid dynamic viscosity *μ*_*f*_ = 0.01 Pa·s. At the initial condition, the fluid is assumed to be at rest and a pressure pulse with peak of 2,000 Pa is imposed at the inlet. The total duration of our observation *T*_max⁡_ for instability is 0.02 s. For majority of the test cases, the instability occurs at the beginning of calculation before reaching *T*_max⁡_. In our program, the fluid acceleration is discretized by the second order accurate backward Euler scheme.

### 4.2. Influence of Simulation Parameters on *γ* versus *ω* Curve

**Figure 5:**
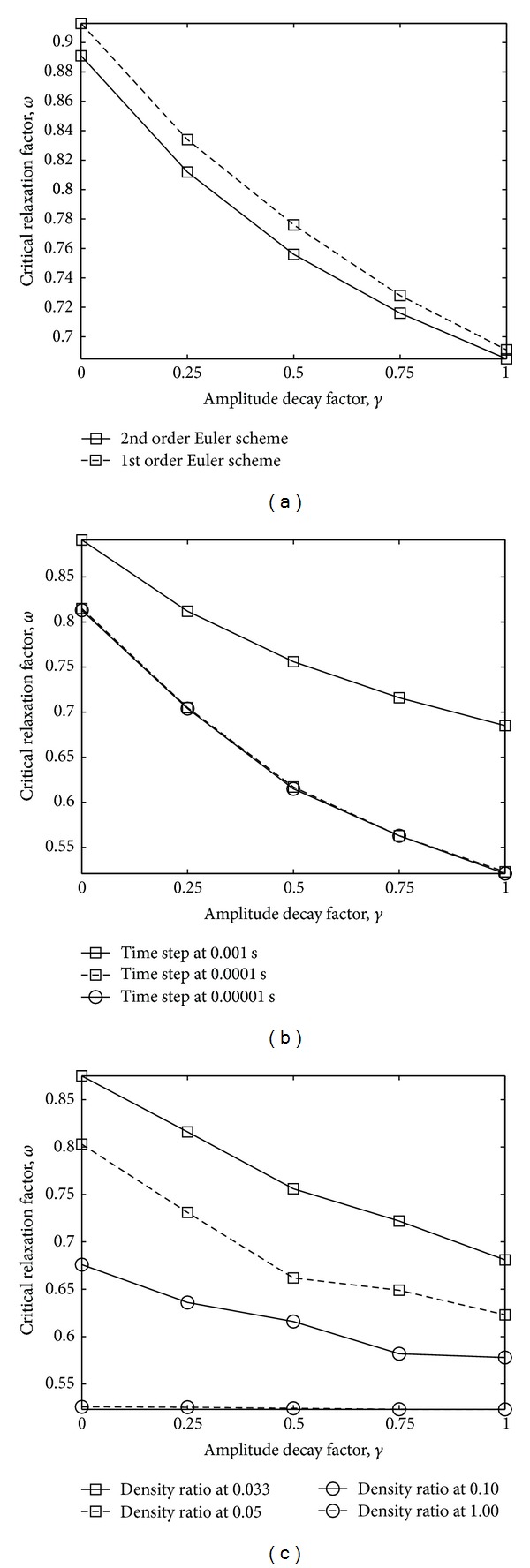
Influence of amplitude decay factor on the critical relaxation factor based on (a) fluid time integration scheme; (b) time step size; and (c) fluid-solid density ratio.

We observe a dependence of the critical relaxation factor *ω* on the amplitude decay factor *γ*. In particular, the required relaxation factors for maintaining stability become greater as the amplitude decay factor approaches zero and the structural time integration scheme becomes the average acceleration method. The numerical tests agree well with our theoretical results, and they confirm that the critical relaxation factor increases with the decrease in the structural amplitude decay factor. Moreover, it is observed that the required values of relaxation factor when using amplitude decay factor of zero can be approximately 30 percent higher than that when using amplitude decay factor of one as shown in Figure 5. By varying parameters and configurations of the FSI architecture such as (a) fluid time integration scheme, (b) time step size, and (c) fluid-solid density ratio, we can obtain information on their effect on the graph of critical relaxation factor versus amplitude decay factor. For all the graphs, it can be demonstrated that the value of critical relaxation factor is inversely proportional to the value of amplitude decay factor.

First, we prove numerically the influence of the fluid time integration scheme on the FSI stability condition. By changing the discretization scheme used for fluid acceleration from the second to the first order accurate backward Euler scheme, the values of critical relaxation factor based on each value of *γ* increase considerably (Figure 5(a)). With *γ* = 1, the solution will be stable if the relaxation factor is set at lower than a conservative value of 0.7, while this threshold increases up to 0.95 when *γ* is set to zero.

Then, the influence of time step size on the stability of FSI calculation is tested. Here, we consider the same domain of calculation and physical parameter as before, and only time step size used for calculation is reduced from 0.001 to 0.0001 s. It is found that time step size also has considerable impact on the performance of FSI simulation. From Figure 5(b), it is shown that the values of critical relaxation factor corresponding with various values of amplitude decay factor decrease considerably when time step size is reduced. Moreover, when the time step size is further reduced to 0.00001 s, the change in critical relaxation factor remains almost unchanged. This observation agrees well with our theoretical results that when the time step size approaches zero, the value of critical relaxation factor converges as presented in Figure 5(b).

The influence of the fluid and solid structure densities on the performance of FSI calculation is considered next. The relationship between fluid-solid density ratio and the critical relaxation factor is based on different values of amplitude decay factor.  Figure 5(c) illustrates the impact that the density ratio has on variation of critical relaxation factor, which is observed to be high only when the density ratio is relatively small. Its gradient tends to vanish as we increase the density ratio value. For fluid-solid density ratio of 0.033, the variation of the critical relaxation factor in the range of *γ* = 0 to 1 is considerably high, while it is negligible at a density ratio of 1.

## 5. Conclusion

We summarize that the stability condition of FSI solution can be influenced significantly by the material densities and the relaxation factor to be implemented. Its computational cost can be greatly reduced by implementation of an appropriate structural time integration scheme. In particular, the critical relaxation factor is higher when using the structural time integration scheme that does not contain numerical damping. Furthermore, the choice of time integration schemes for discretizing fluid acceleration has a strong impact on the stability condition. Our results suggest that more accurate schemes such as the second order accurate backward Euler can lead to a more stringent condition for the stability of FSI. Another important parameter is the time step size. Then, smaller time step size results in a more restrictive condition, and as the time step size approaches zero, the value of the critical relaxation factor converges to a specific value. Another factor worth mentioning is the density ratio of fluid to solid structure. It is found that this value has a considerable impact on FSI performance, and the stability condition is invariant to the choice of structural time integration schemes in the case of high fluid-solid density ratios. However, for the low density ratios, this value will mainly depend on the schemes used.
